# Target DNA Structure Plays a Critical Role in RAG Transposition

**DOI:** 10.1371/journal.pbio.0040350

**Published:** 2006-10-24

**Authors:** Jennifer E Posey, Malgorzata J Pytlos, Richard R Sinden, David B Roth

**Affiliations:** 1 Department of Molecular and Human Genetics, Baylor College of Medicine, Houston, Texas, United States of America; 2 Program in Molecular Pathogenesis, Skirball Institute of Biomolecular Medicine, and Department of Pathology, New York University School of Medicine, New York, New York, United States of America; 3 Laboratory of DNA Structure and Mutagenesis, Center for Genome Research, Institute of Biosciences and Technology, Texas A&M University System Health Sciences Center, Houston, Texas, United States of America; Scripps Research Institute, United States of America

## Abstract

Antigen receptor gene rearrangements are initiated by the RAG1/2 protein complex, which recognizes specific DNA sequences termed RSS (recombination signal sequences). The RAG recombinase can also catalyze transposition: integration of a DNA segment bounded by RSS into an unrelated DNA target. For reasons that remain poorly understood, such events occur readily in vitro, but are rarely detected in vivo. Previous work showed that non-B DNA structures, particularly hairpins, stimulate transposition. Here we show that the sequence of the four nucleotides at a hairpin tip modulates transposition efficiency over a surprisingly wide (>100-fold) range. Some hairpin targets stimulate extraordinarily efficient transposition (up to 15%); one serves as a potent and specific transposition inhibitor, blocking capture of targets and destabilizing preformed target capture complexes. These findings suggest novel regulatory possibilities and may provide insight into the activities of other transposases.

## Abstract 2

Hairpin structures are preferential targets for RAG-mediated DNA transposition. However, the presence of a CT dinucleotide at the hairpin tip strongly inhibits transposition.

## Introduction

The normal gene-rearranging activities of V(D)J recombination bear a striking resemblance to transposition by certain “cut-and-paste” transposases, including members of the retroviral integrase superfamily [1–3] and the *hAT* family [4]. In both reactions, a segment of DNA bounded by specific recognition elements is excised from the chromosome [1,2]. The key difference is that in transposition, the transposase inserts the excised fragment elsewhere in the genome, whereas in V(D)J recombination, the excised segment is typically lost from the cell [1,2]. Purified RAG proteins can, however, transpose a DNA sequence flanked by two conserved sequence elements (recombination signal sequences or RSS) differing in length (i.e., a 12-RSS and a 23-RSS) into an unrelated DNA target [5,6]. This discovery led to new models for RAG involvement in many of the as-yet unexplained oncogenic translocations involving immunoglobulin and T cell receptor loci [2,6–8]. To date, however, few in vivo rearrangements have been unequivocally identified as RAG transposition events [9–11]. In light of this, investigators have proposed a variety of potential regulatory controls that might limit transposition, such as reversal of the reaction by disintegration [7] or inhibition of transposition by the C-terminus of RAG2 [12–14] or by GTP [13].

Given that some transposases show distinct preferences for certain targets, it was reasonable to hypothesize that target site selectivity could serve as a mechanism to curb RAG-mediated transposition without affecting RAG recombination activity [15]. Insertion into preferred target sites could steer transposition events to innocuous genomic locations [15,16] or into the formation of harmless alternative V(D)J recombination products termed “open-and-shut” and “hybrid” joints [16]. Early studies suggested that RAG transposition events, although detected throughout plasmid target sequences, favor GC-rich regions [5,6]. A subsequent study of intramolecular transposition reported a modest preference for a GC-rich hotspot sequence located near the center of a short (329 base pair [bp]) excised fragment [17], although the short length of the target and the limited flexibility of short duplex DNA fragments would restrict the range of other available target sequences in this situation. More recent experiments showed that transposition is stimulated by targets bearing hairpin ends; even in a relatively large (2,700 bp) plasmid target, the overwhelming majority of transposition events (84%) occurred within the terminal four nucleotides of the hairpin tips of a cruciform structure [16]. Subsequent work confirmed that cruciforms are preferred targets [11].

Given that the terminal four nucleotides of a hairpin strongly influence recognition by structure-specific endonucleases [18], we sought to determine how the sequence of the terminal nucleotides of a hairpin might influence the efficiency of transposition. We investigated the ability of oligonucleotide hairpin targets bearing each of 16 possible self-complementary four-nucleotide hairpin tip sequences to stimulate transposition, and we also analyzed several different cruciform tip sequences in the context of supercoiled plasmids. Our data show that certain hairpin sequences stimulate extraordinarily efficient transposition (15% of substrate converted to product), revealing the RAG transposase to be far more active than previously suspected. We also discovered that one particular hairpin serves as a potent and specific inhibitor of transposition, impairing target capture and/or destabilizing already formed target capture complexes. This mechanism provides a potential regulatory control that acts at a specific step that is essential for transposition, but dispensable for normal V(D)J recombination.

## Results

### Hairpin Tip Sequence Modulates Transposition Efficiency

**Figure 1:**
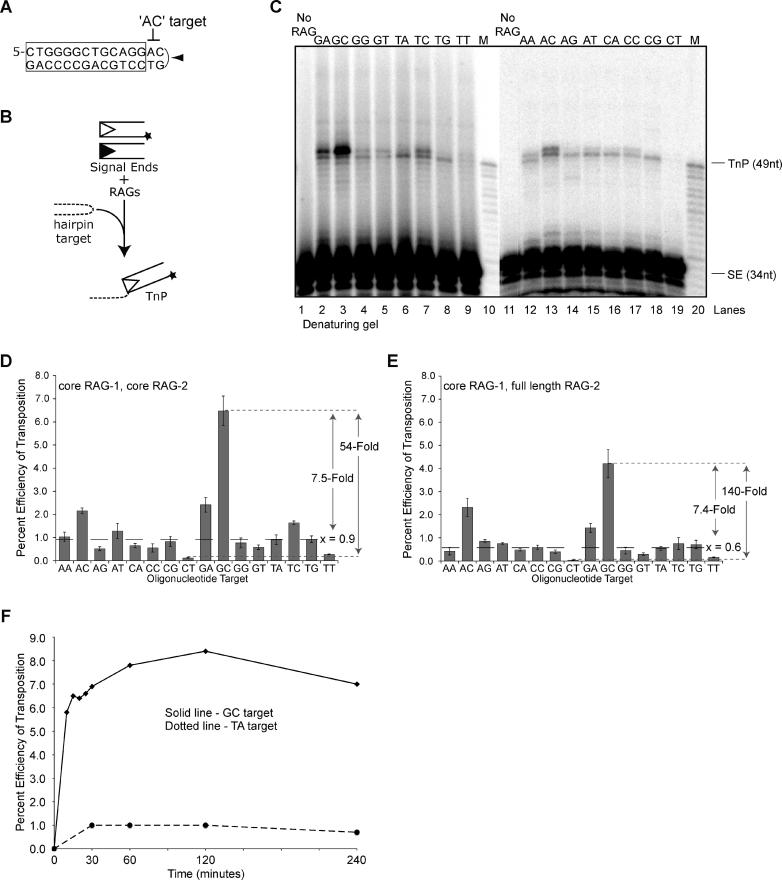
Hairpin Tip Sequence Modulates Transposition Efficiency (A) The complete sequence of hairpin “AC,” one of the 16 oligonucleotide-encoded inverted repeat targets tested. All inverted repeats fold into hairpins sharing the same stem sequence (box). Hairpins differ only in the four nucleotides surrounding the dyad axis of the inverted repeat (arrowhead) and are named according to the first two nucleotides of this sequence (bold). (B) Schematic of typical in vitro transposition reaction. Transposition products are formed from covalent attachment of P^32^-labeled (star [★]) signal ends to hairpin targets by RAG complex. (C) Hairpin sequence affects efficiency of transposition. Following a 30-min reaction, transposition products are separated on a 15% sequencing gel. (D) and (E) Hairpin sequences are differentially targeted by the RAG transposase using both core RAG1/core RAG2 (D) and core RAG1/full-length RAG2 (E) preparations. Graphs depict efficiency of transposition into each hairpin target after a 30-min reaction, averaged over three experiments with two separate RAG protein preparations. Error bars represent standard error of the mean. “x” indicates the median value for the 16 hairpin tips. (F) Disintegration does not explain the variation in hairpin targeting. Graph depicts time course of transposition into the GC (solid line) and TA (dotted line) targets. At time points earlier than 30 min, bands representing transposition products formed from the TA target were not substantial enough for quantification. Transposition products formed from the CT target were also prepared at the same time points; there was not sufficient product for quantification. Data for (D), (E), and (F) obtained using PhosphoImager and quantified using ImageQuant software. M, DR117TnRef, reference oligonucleotide used as a size marker for transposition products; No RAG, mock reaction lacking RAG proteins; SE, signal ends; TnP, transposition products; star (★), P^32^ 5'-end label.

The hairpin structures previously examined bore the tip sequence 5′ TA • TA 3′ in a variety of different stem configurations [16]. To determine whether hairpins with other sequences at the tip might also act as preferred targets for RAG transposition, we designed a set of 16 self-complementary oligonucleotides with all possible four-nucleotide combinations surrounding the dyad axis of the inverted repeat. Each of these oligonucleotides bears the same stem sequence and should anneal into a double-stranded molecule bearing one hairpin end. The sequence of the stem is shown in Figure 1A, in this case terminating in the AC hairpin tip. We tested the ability of these hairpin oligonucleotides to serve as targets for in vitro transposition by incubating them with core RAG proteins and precleaved RSS (signal ends) in a standard transposition assay (in Mg^2+^) (Figure 1B), followed by denaturing gel electrophoresis. Most transposition events occurred at or very near the hairpin tips (Figure 1C), in agreement with previous work [16].

To quantify the effects of different hairpins, we calculated the transposition efficiency for each target as the percentage of signal ends transposed into that target. The sequence of the hairpin tip modulated the efficiency of transposition over a more than 50-fold range, from virtually undetectable (the CT hairpin) to quite robust (>6% for the GC hairpin) (Figure 1C, lanes 19 and 3, respectively; Figure 1D). The TA hairpin (Figure 1C, lane 6), corresponding to the tip sequence studied previously [16], yielded a transposition efficiency near the median (0.9%) calculated for all 16 hairpins. One hairpin sequence, CT, was an especially poor target, with values lower than non-hairpin targets (unpublished data; also see below). These results show that the nucleotide sequence, rather than the base composition of the tip, is critical: TC, for example, gave at least 10-fold more transposition than CT (Figure 1D). In light of earlier work, it is particularly interesting to note that GC was at least 7-fold more active than CG; this is one clear indication that features other than GC content are important for transposition (see below).

Since the C-terminus of full-length RAG2 (which is lacking from the core version of the protein) has been implicated in down-regulating transposition activity [12–14], we repeated our measurements of transposition into all 16 hairpin sequences using full-length RAG2 (Figure 1E). The data generally followed the trends established by core RAG2. Although a few hairpin sequences showed a decrease in transposition efficiency, reducing the median transposition efficiency across all sequences from 0.9% to 0.6%, the range of efficiencies was even greater than that observed with core RAG2, with a 140-fold difference between the most active (GC) and the least active (CT) target sequences. Note that the efficiency of transposition into the GC hairpin remained roughly 7.5-fold more than the median, as observed for core RAG2. We conclude that, with precleaved ends and hairpin targets, the C-terminus of RAG2 does not substantially suppress transposition activity and does not affect the choice of hairpin tip sequences used as transposition targets.

Transposition products can be “disintegrated” in vitro by a reversal of the transposition reaction: the RAG proteins can catalyze a nucleophilic attack on the phosphate backbone linking the signal end to target, either through hydrolysis or transesterification, using the 3′-hydroxyl on the target created by the initial transposition event [7]. High levels of Mg^2+^ (25 mM) are optimal for disintegration in vitro [7]. Although all of our reactions were performed at more physiologic Mg^2+^ levels (3 mM), we considered the possibility that differences in apparent transposition efficiency might be influenced by sequence-specific effects on disintegration. To test this hypothesis, we performed time course experiments using several hairpin tip sequences: CT, TA, and GC. With TA and GC, we did not observe any decrease in the levels of products prior to the 2-h time point (Figure 1F), suggesting that disintegration did not play a major role in determining levels of transposition products during the 30-min incubation in Figure 1C–1E. (Transposition into CT was too low to be detected at any time points in this experiment.) We conclude that the hairpin tip sequence exerts its primary effect on the formation rather than the stability of transposition products.

Because self-complementary oligonucleotides can exist in two stable base-paired isoforms, hairpins (formed by intra-molecular annealing) or dimers (formed by inter-molecular annealing) (Figure S1A), additional control experiments were necessary. We examined three annealed targets (the least active target, CT, and two average targets, TA and TG) by native gel electrophoresis to resolve the hairpin and dimer isoforms, both before and after a transposition reaction. Both the CT and TA hairpin targets annealed to form hairpins and remained exclusively hairpins under our conditions; only TG showed a mild tendency to form dimers (Figure S1B and S1C). All three targets yielded the expected transposition products (Figure S1D). Based on these data as well as experiments using supercoiled plasmids bearing extruded cruciforms (see below), the variations in transposition efficiency observed in Figure 1 are not attributable to tip sequence effects on hairpin formation or stability.

We next tested how much influence the sequence distal to the four terminal nucleotides of the hairpin might have on the efficiency of transposition by altering either the entire stem sequence of the hairpin or simply the nucleotide in the third position (n-3) from the dyad axis of the inverted repeat (Figure S2A and S2B). Neither change had any significant effect on the RAG transposase's preferences (Figure S2C and S2D). These data confirm that the sequence of the four nucleotides around the hairpin tip is the primary determinant of the ability of the RAG transposase to employ the hairpin as a target.

### Cruciform-Bearing Plasmid Targets Also Show Strong Sequence Effects

**Figure 2:**
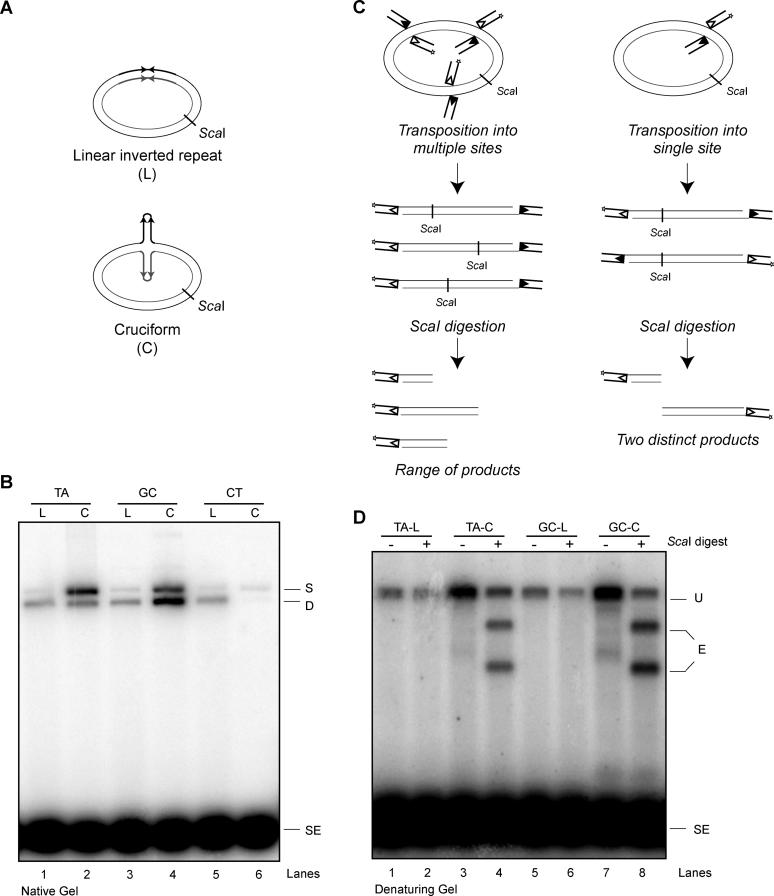
Transposition Is Targeted to Structures, Not Sequences (A) Depiction of two plasmids encoding inverted repeats, identical in sequence. One plasmid stably maintains a linear inverted repeat; the other forms a stable cruciform, with two extruded hairpin arms. (B) Transposition is stimulated by cruciform structures. Single insertion (nicked plasmid) and double insertion (linearized plasmid) transposition products formed from three inverted repeat targets in linear or cruciform conformation were resolved on a 1% native agarose gel. Results shown here are representative of three independent experiments; similar data were obtained with a second RAG protein preparation. (C) ScaI digestion of plasmids after transposition yields two distinct products when transposition is targeted to a specific part of the plasmid. A plasmid lacking cruciforms is used for illustration. (D) Plasmid-encoded inverted repeats target transposition only when a cruciform is extruded. After digestion with ScaI, transposition products were separated on a 1% alkaline agarose gel. C, cruciform; D, double insertion product; E, expected sizes of digested products if transposition was targeted to cruciform; L, linear inverted repeat; S, single insertion product; SE, signal ends; U, undigested transposition products.

To evaluate the effects of hairpin tip sequence on transposition efficiency using more physiologic substrates, we constructed three plasmids encoding fully self-complementary inverted repeats that differ only in the four nucleotides surrounding the dyad axis of the repeat. This allowed us to place the hairpin sequences in direct competition with the 2,700-bp plasmid backbone as transposition targets. Under conditions of sufficient negative supercoiling, the 106-bp inverted repeat sequences form a stable cruciform structure composed of two 53-bp cruciform arms [19,20] (Figure 2A). A series of DNA topoisomer preparations of various superhelical densities was made [21–23]. Upon analysis by two-dimensional gel electrophoresis (unpublished data), a DNA sample in which 100% of the plasmids contained fully extruded cruciforms was used for transposition studies. As a negative control, a relaxed plasmid was prepared so that the inverted repeat would remain in the linear conformation. Each of the three plasmids was tested with the inverted repeat in the linear conformation, and, in agreement with previous work [16], each supported low levels of transposition (Figure 2B, lanes 1, 3, and 5). In the supercoiled sample containing 100% cruciforms, hairpins containing the TA and GC tips stimulated transposition (lanes 2 and 4), whereas the CT hairpin remained a very poor target (lane 6). The GC cruciform was a better target than TA, and we observed a shift toward more double insertion products with this target. GC stimulated up to 100-fold more transposition events than CT (Figure 2B, compare lanes 4 and 6).

To determine whether transposition events in these plasmids occurred at the site of the inverted repeat or elsewhere, we took advantage of a unique ScaI restriction site in the plasmid (Figure 2A). If transposition events occurred at the inverted repeat, ScaI digestion would yield two discrete, labeled products of predictable size under denaturing conditions (to measure both single and double insertion events), but random transposition events throughout the target would yield a smear of labeled products (Figure 2C). ScaI digestion did in fact yield two distinct products, but only when the cruciforms were extruded (Figure 2D). (Note that even though the ScaI digestion was incomplete, more than 95% of the digestion products appeared in the two expected fragments, indicating that transposition is consistently targeted to the hairpin ends.) Our results clearly show that transposition is targeted to cruciform structures that compete very effectively with the 2.7-kb duplex DNA flanking the cruciforms as transposition targets (Figure 2D). The marked difference in the efficiency with which the different cruciform tip sequences were targeted provides further evidence that the preference of the RAG transposase for a given hairpin is significantly influenced by the nucleotides at the tip.

### Hairpins Target Significantly More Transposition than a GC-Rich “Hotspot”

**Figure 3:**
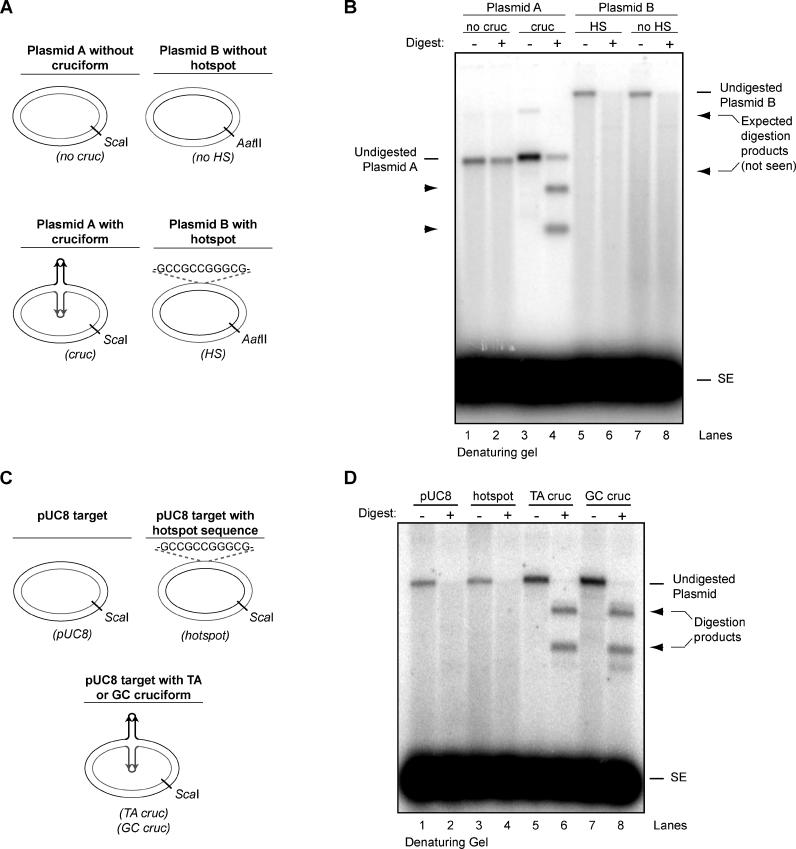
The RAG Transposase Prefers Hairpin Structures to GC-Rich Target Sequences (A) Diagram of plasmid targets. (B) A plasmid-encoded cruciform structure with an average hairpin tip (TA) stimulates much greater levels of RAG-mediated transposition than a previously defined 11-bp GC-rich target sequence. Transposition products were subjected to ScaI (Plasmid A) or AatII (Plasmid B) digestion before separation on a 1% alkaline agarose gel. Targeted transposition yields two distinct products (arrowhead), whereas non-targeted transposition yields a smear of labeled products. (C) Diagram of three targets with identical plasmid backbones. (D) TA and GC cruciforms stimulate much greater levels of RAG-mediated transposition than a GC-rich hotspot sequence in an identical plasmid backbone. Transposition products were subjected to ScaI digestion before separation on a 1% alkaline agarose gel. cruc, Plasmid A with cruciform; GC cruc, GC inverted repeat extruded from pUC8 backbone; hotspot, GC-rich hotspot sequence cloned into pUC8 backbone; HS, Plasmid B with 11-bp GC-rich hotspot sequence; no cruc, Plasmid A without cruciform; no HS, Plasmid B without 11-bp GC-rich hotspot sequence; SE, signal ends; TA cruc, TA inverted repeat extruded from pUC8 backbone.

We next compared one of our hairpin targets to the only specific sequence heretofore reported to represent a preferred target for RAG transposition: 5′-GCCGCCGGGCG, a “hotspot” originally discovered in the context of intramolecular transposition, in which the signal ends must insert near the center of a short (329 bp) linear DNA fragment for steric reasons [5,17]. We initially compared this hotspot in its original plasmid context (Plasmid B, Figure 3A) to a plasmid containing an extruded TA cruciform (Plasmid A, Figure 3A). The plasmid containing the GC-rich hotspot was no better a target than an identical plasmid backbone without the GC-rich sequence (Figure 3B, compare lanes 5 and 7), and no targeted events were detected after digestion (lanes 6 and 8). In contrast, Plasmid A, containing an average TA cruciform, stimulated more transposition than the same plasmid lacking the cruciform (compare lanes 1 and 3), and transposition was targeted to the tips of the cruciform (lane 4).

In order to compare several targets sharing the same plasmid backbone, we cloned the GC-rich sequence into the pUC8 plasmid (Figure 3C). We then compared a pUC8 plasmid containing an extruded GC cruciform (our best target), as well as one containing a TA cruciform, to an identical plasmid containing this 11-bp GC-rich sequence (Figure 3C). As expected, both cruciform structures strongly stimulated transposition (Figure 3D, compare lanes 5 and 7). In contrast, the GC-rich sequence did not noticeably affect the efficiency of transposition into the plasmid in comparison with an identical pUC8 plasmid lacking the GC-rich sequence (Figure 3D, compare lanes 1 and 3). Digestion of the transposition products with a single-cutting restriction enzyme revealed no evidence that transposition events were targeted specifically to the vicinity of the GC-rich sequence (Figure 3D, lane 4). This is in sharp contrast with the cruciforms (Figure 3D, lanes 6 and 8). Thus, even an average cruciform target can provide a significantly more attractive target for transposition than the previously reported GC-rich “hotspot” sequence.

### A Novel Transposition Inhibitor

As shown in Figure 2, the RAG transposase targeted plasmids containing linear inverted repeats with low efficiency. This efficiency rose dramatically when inverted repeats were extruded as cruciforms, with one interesting exception. Upon extrusion of the CT cruciform, transposition efficiency actually *decreased* from already-low levels by approximately 90% (Figure 2B, compare lanes 5 and 6). This result suggested that the CT hairpin could be suppressing transposition events elsewhere in the plasmid.

**Figure 4:**
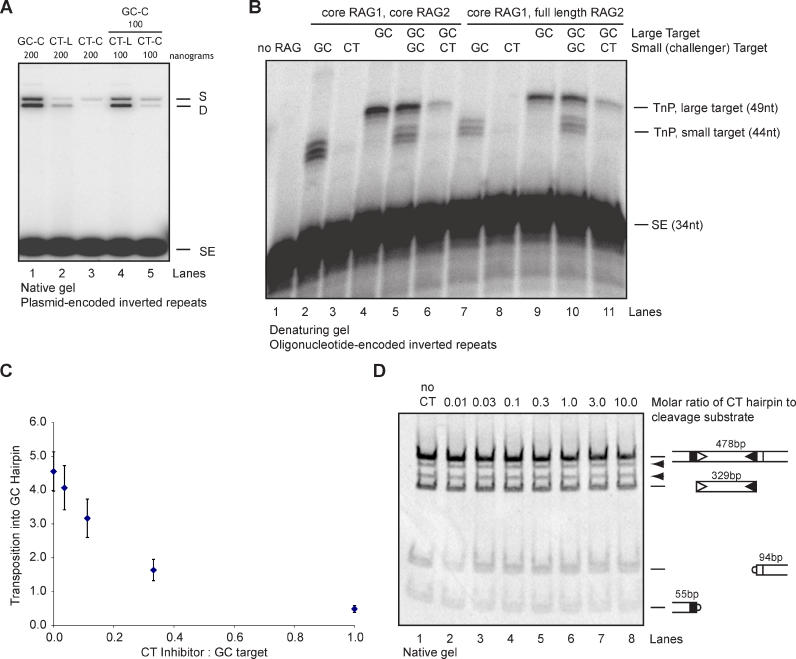
The CT Hairpin Inhibits Transposition (A) CT cruciform inhibits transposition into a plasmid target. Transposition was performed using a GC cruciform target mixed with either the linear CT inverted repeat or CT cruciform (lanes 4 and 5). Amount of each plasmid used is indicated. (B) CT oligonucleotide-encoded inverted repeat inhibits transposition into an oligonucleotide target. A total of 0.5 pmol of hairpin target or a mix of 0.25 pmol each of two targets was added to a transposition reaction (lanes 5 and 6). Reaction was performed using both core RAG2 and full-length RAG2 preparations. Transposition products were resolved on a 15% denaturing acrylamide gel. Small GC and CT hairpins are GC-2 and CT-2, as described in Figure S2A. (C) CT hairpin is a potent inhibitor. Experiments were performed using oligonucleotide-encoded hairpins. Error bars represent standard error of the mean calculated from three independent experiments. (D) CT hairpin does not inhibit cleavage of a PCR-generated substrate, even at 10-fold molar excess. Cleavage substrate was mixed with increasing amounts of CT hairpin prior to addition of RAG protein. Cleavage products were separated on a native 4%–20% acrylamide gradient gel. Expected cleavage products are indicated to right of gel; arrowheads indicate single cleavage products. C, cruciform; D, double insertion product; L, linear inverted repeat; no CT, CT hairpin was not added to reaction; no RAG, mock reaction lacking RAG proteins; S, single insertion product; SE, signal ends; TnP, transposition products. Oligonucleotide hairpins are as described in Figure 1A.

To pursue this observation, we set up a competition experiment using equal masses of two plasmid targets of equivalent length: GC cruciform and either CT cruciform or CT linear inverted repeats. The CT cruciform substantially inhibited transposition (>90%) (Figure 4A; compare lanes 1 and 5). In contrast, the linear CT target had no effect on transposition into the GC cruciform (compare lanes 1 and 4). This result was confirmed and extended by a competition experiment using oligonucleotide targets (Figure 4B). In this experiment, transposition into a preferred GC hairpin (lane 4) was challenged by addition of a smaller GC target (lane 2 shows transposition products formed from this target alone) or a smaller CT target (lane 3 shows transposition products formed from this target alone). Addition of an equimolar amount of the CT hairpin oligonucleotide almost completely (>90%) suppressed the usually robust transposition targeted to a GC hairpin (compare lanes 4 and 6). This effect was in stark contrast to challenge of the GC target by a different (smaller) GC target (lane 5); in this case, the smaller target had little effect on transposition into the larger target. As expected, total transposition into the GC targets in lane 5 was quantitatively similar to that seen in lane 4. We conclude that inhibition of transposition is a specific effect of the CT hairpin. These findings were reproduced using full-length RAG2 preparations (Figure 4B, lanes 7–11). The CT hairpin also inhibited transposition into an AC hairpin, indicating that this effect is not specific to the GC target (unpublished data). Furthermore, we note a 90% reduction of transposition into the 2,700-bp plasmid backbone in the presence of a 106-bp CT cruciform, suggesting that inhibition of transposition by a CT hairpin is not specific to hairpin targets (Figure 2B, compare lanes 5 and 6).

To assess the potency of the inhibitor, we examined the effect of decreasing amounts of inhibitor on transposition efficiency. At only one-fifth the concentration of the GC target, the inhibitor reduced transposition by approximately 50% (Figure 4C). We conclude that the CT cruciform is a strong inhibitor of transposition both in *cis* (in the same target molecule) and in *trans*.

The inhibitory effects described above could not be attributed to effects on cleavage, because the protocol used in these experiments employs precleaved donor RSS oligonucleotides. Since the RAG proteins catalyze transposition by an intermolecular transesterification mechanism, we next assessed the effect of the inhibitor sequence on the cleavage step, which involves intramolecular transesterification (hairpin formation). We added increasing amounts of the CT hairpin to a standard cleavage reaction using molar ratios of inhibitor:recombination substrate of up to 10:1. No inhibition of double-strand break formation was observed, indicating that the inhibitor sequence does not affect nicking or hairpin formation (Figure 4D). We also tested the ability of the RAG complex to make hairpins from a pre-nicked substrate in the presence of the inhibitor (Figure S3A). Neither the CT hairpin inhibitor sequence nor the GC control sequence affected hairpin formation (Figure S3B). These data demonstrate that the CT hairpin specifically inhibits transposition.

**Figure 5:**
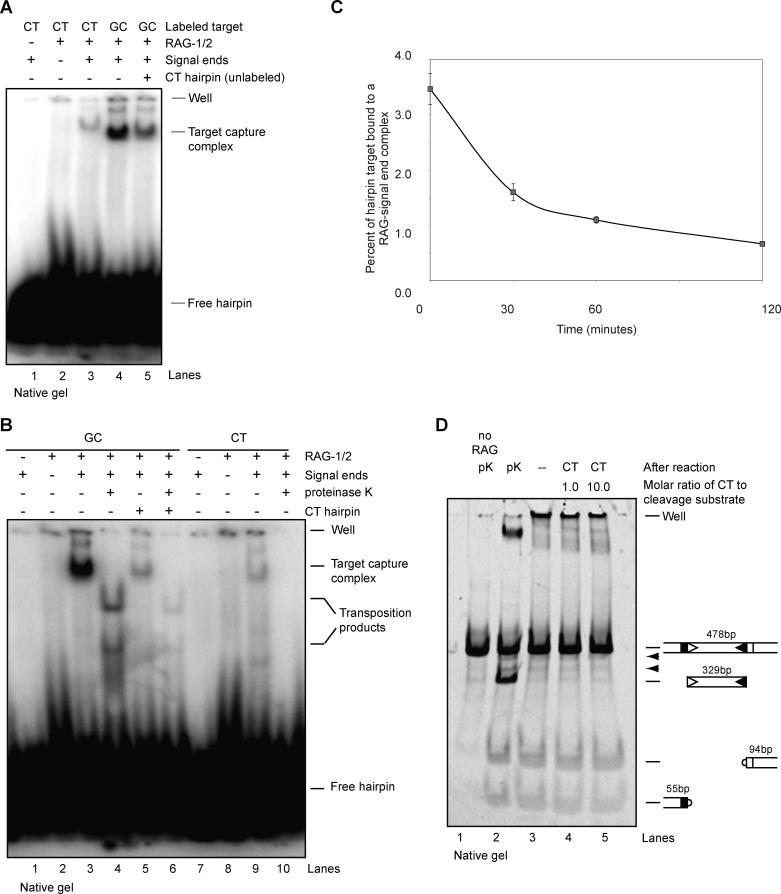
The CT Hairpin Inhibits Target Capture (A) CT hairpin is bound weakly by the RAG–signal end complex and inhibits target capture of a GC target. Using labeled CT or GC target, target complex formation was detected on a native 4%–20% gradient acrylamide gel (lanes 3 and 4). Unlabeled CT hairpin was mixed in a 1:1 molar ratio with labeled GC target prior to start of reaction (lane 5). (B) GC target is bound by the RAG-signal end complex. CT hairpin is bound only weakly by the RAG–signal end complex and inhibits target capture of a GC target. Using labeled GC or CT target, complex formation was detected on a native 4%–20% gradient acrylamide gel. Proteinase K treatment indicates amount of RAG-bound transposition products present in DNA-protein complex. Unlabeled CT hairpin was mixed with labeled GC target prior to start of reaction (lanes 5 and 6). (C) CT hairpin destabilizes preformed GC target capture complexes. Radiolabeled GC target was bound to RAG–signal end complexes for 120 min, upon which non-labeled CT inhibitor was added to reaction. Reaction was stopped after an additional 30, 60, or 120 min, as indicated. (D) CT hairpin does not destabilize signal end complexes formed by cleavage. RAG proteins were incubated for 3 h with PCR-generated cleavage substrate. After the reaction, either stop buffer (containing proteinase K and SDS) or CT inhibitor was added. Expected cleavage products are indicated to right of gel; arrowheads indicate single cleavage products.

To probe the mechanism of inhibition, we assessed target capture of hairpin oligonucleotides by the RAG transposase. Previous work demonstrated that the RAG proteins form a target capture complex, containing target DNA and a 12/23-RSS pair, that is stable to polyacrylamide gel electrophoresis [16]. These results were recapitulated using a precleaved 12/23-RSS pair and a radiolabeled hairpin target in Figure 5A; the RAG–signal end complex exhibited robust binding to the GC hairpin (lane 4). In contrast, binding to a CT hairpin was less efficient (Figure 5A, lane 3). In spite of its weak interaction with the RAG–signal end complex, the CT hairpin substantially diminished target capture of the GC hairpin target when present in a 1:1 molar ratio (lane 5). This effect was not observed with control non-hairpin or with noninhibitory hairpin sequences (GC, TA) present at 1:1 molar ratios (unpublished data). These findings suggest that the CT hairpin sequence is able to inhibit or destabilize target interactions with the RAG–signal end complex.

The DNA–protein complexes detected in electrophoretic mobility shift experiments such as those described above consist of two species: a target capture complex (RAG proteins, signal ends, and target) and a strand transfer complex containing the same components, but with a covalent linkage between the signal ends and the target DNA [16]. To distinguish between these species, we split reactions into two equal parts and subjected them to gel electrophoresis with and without deproteinization (in the former case, to determine what fraction of the DNA–protein complexes correspond to covalently linked transposition products). In agreement with the data shown in Figure 5A, the CT inhibitor decreased capture of the GC target (Figure 5B, compare lanes 3 and 5). Deproteinization revealed that about 20% of the DNA–protein complexes with the GC target consists of transposition products (Figure 5B, compare lanes 3 and 4). We detected no transposition products with the CT hairpin (lane 10). As expected, the CT hairpin also markedly decreased levels of transposition products formed from the GC target (compare lanes 4 and 6).

Could the inhibitor sequence destabilize preformed target capture complexes? We added unlabeled CT hairpin (at a 1:1 molar ratio with respect to labeled GC target) and assessed its effect on target capture complexes over time (Figure 5C). The amount of target capture complex diminished substantially over the observed period, with only one-fifth the original amount remaining after 2 h (Figure 5C); this decrease is similar to that observed in transposition products in experiments described earlier (Figure 4C). These data indicate that the CT hairpin sequence destabilizes preformed target capture complexes.

How might this destabilization take place? The CT hairpin might either destabilize target binding or disrupt the RAG–signal end complex. We therefore examined the effect of the CT hairpin on signal end complexes using an electrophoretic gel mobility shift assay that measures release of free signal ends. In this experiment, the RAG–signal end complexes generated by cleavage were not destabilized by the CT hairpin, even when it was present in a 10-fold molar excess over the signal ends (Figure 5D). These data, along with the failure of the inhibitor sequence to affect cleavage (see above), demonstrate that the effect of the CT hairpin is specific to target (and not RSS) binding.

Finally, we considered the possibility that the inhibitor sequence might stimulate removal of transposition products by disintegration. To test this, we generated oligonucleotide substrates corresponding to transposition products that would be derived from the GC hairpin and incubated these in the presence of RAG proteins and buffer, GC hairpin, or CT hairpin sequences. The results clearly show that neither of the hairpin sequences stimulated disintegration (Figure S3).

## Discussion

### Hairpins Can Stimulate Surprisingly Efficient RAG Transposition

Our data show that most hairpins are strongly attractive targets for RAG transposition and that the efficiency of target utilization is profoundly modulated by the sequence of the terminal nucleotides of the hairpin. Of the 16 possible tip sequences, the majority were strong targets for the RAG transposase in the context of self-complementary oligonucleotides. When we tested a subset of these sequences as both cruciforms (in supercoiled plasmids) and as linear inverted repeats (in relaxed plasmids), only the cruciform structures stimulated transposition. Finally, we found that even an average hairpin target sequence (TA) stimulated far more transposition activity than a previously reported GC-rich hotspot.

When RAG transposition was first discovered, it was noted that there was a preference for GC-rich sequences [5,6]. A subsequent analysis of intramolecular transposition reported a modest preference for a GC-rich hotspot sequence located near the center of a short (329 bp) excised fragment [17], although the range of other available target positions in this molecule was restricted by steric constraints (the short length of the linear DNA) in this setting. The most likely explanation for the reported GC-rich sequence preference is that these base pairs are more likely to form altered DNA structures. Nevertheless, our data show that the GC content alone is not the determining factor in targetability: the GC cruciform was a significantly better target than CG, CC, or GG. Tsai et al. also compared four self-complementary oligonucleotide sequences with three different tips (CT, TG, and two TA tips) and reported that only one TA tip was a preferred target for the RAG transposase, although no quantification was provided [17]. Our data agree that the CT tip is not a transposition target for the RAG proteins. It is not clear why the authors failed to detect transposition with the TG or second TA tip; it is possible that these two oligonucleotides did not form hairpins under the annealing conditions used in that study.

### The Sequence of the Hairpin Tip Strongly Affects Its Utilization as a Target for Transposition

Transposition efficiency among the 16 oligonucleotide hairpins varied over a 50- to 140-fold range and was determined primarily by the sequence of the four nucleotides around the hairpin tip. This variation was not secondary to disintegration. The most attractive target, a hairpin with the terminal sequence GC, was surprisingly efficient, and even more so (15%) under our most physiologic in vitro transposition conditions (a plasmid target, in Mg^2+^). These results are especially striking in light of the fact that the in vitro cleavage efficiency of our RAG protein preparations under these conditions is generally 20%–30%. This provides the first evidence that a substantial fraction of post-cleavage signal end complexes is actually capable of transposition. This result is unexpected, given the prevailing opinion that RAG proteins are, in general, a rather inefficient transposase. The exception to the rule—the CT hairpin, which specifically inhibits transposition without affecting RSS cleavage—is no less surprising and is discussed in more detail below.

### How Does Hairpin Tip Sequence Influence Transposition?

How does the sequence of the hairpin tip so strongly affect its use as a target? Since all hairpins tested remain largely folded as hairpins (and the cruciforms are very stable under our experimental conditions [19]), targeting to hairpin ends does not appear to be secondary to DNA melting, as was previously suggested [17]. Our data lead us to propose an alternative hypothesis: the strong effect of hairpin sequence on transposition efficiency is driven by sequence-induced structural differences at the various hairpin tips. Two lines of evidence support this model. First, hairpin sequence affects recognition by structure-specific nucleases [18,24]. Second, nuclear magnetic resonance (NMR) studies have shown that the sequence at a DNA hairpin tip significantly affects its three-dimensional structure [25,26]. Typically, stable self-complementary loops consist of either two or four nucleotides [25,26]. Some evidence suggests that many tips having a pyrimidine in the second nucleotide position 5′ of the dyad axis and a purine in the second nucleotide position 3′ of the axis (x _ • _ x) form loops of two, not four, nucleotides [27,28]. Indeed, our most poorly used hairpin targets, CT and TT, fit this description, suggesting a possible correlation between loop size and the ability of the RAG complex to target a given hairpin.

### A Specific and Potent Inhibitor of Transposition

We were intrigued by the CT hairpin tip sequence that is refractory to transposition. We considered two potential explanations for this result: the RAG transposase may fail to bind to the CT tip structure(s), or it may bind in a nonproductive mode that impedes catalysis. A prediction of the second model is that the CT hairpin should inhibit transposition into other substrates, which is indeed what we observed. The CT sequence, when in hairpin form, inhibited transposition into the plasmid backbone and even into the most favorable target, the GC hairpin. It is interesting to note that the CT hairpin is a much more potent inhibitor than the only other previously reported inhibiting factor, GTP: with the hairpin, nearly complete inhibition is obtained at a 1:1 inhibitor to target ratio, as opposed to the greater than 10,000:1 ratio for GTP [13]. Our data suggest that the CT hairpin tip binds the transposase in some fashion that renders the complex refractory to transposition. That the RAG–signal end complex is able to bind the CT hairpin, albeit weakly, supports this possibility. The fact that the CT hairpin rather inefficiently forms a target capture complex indicates that this DNA structure may bind in a different mode. Investigation of the precise mode and site of such binding awaits the development of more sophisticated analytical techniques.

### Mechanism of Inhibition

Since our data indicate that the inhibitor does not affect DNA cleavage (hydrolysis and intramolecular transesterification), the CT hairpin likely does not affect the catalytic step of transposition. We also found that it does not stimulate disintegration. This inhibitor does, however, impede the ability of the RAG proteins to efficiently capture target, and it destabilizes preformed target capture complexes without affecting the stability of signal end complexes. These findings suggest a plausible mechanism for inhibition: we propose that the inhibitor specifically prevents stable target capture, inhibiting transposition without affecting DNA cleavage (or, presumably, the subsequent steps in V(D)J recombination).

Given that hairpins are preferred targets, it is reasonable to hypothesize that the portion of the active site responsible for binding to the hairpin coding ends in the postcleavage complex could serve as the target binding site. It is not known when this binding pocket is formed during the course of the reaction. There are at least three distinct possibilities: (1) the target binding pocket is present before cleavage (and likely consists of the coding flank binding site); (2) it corresponds to a portion of the active site that stabilizes a “pre-hairpin” transition state that is present prior to completion of cleavage, but perhaps not at the beginning of the reaction; or (3) the target binding site is revealed only after cleavage, perhaps by a conformational change induced by strong binding of the RAG proteins to the signal ends forming the stable signal end complex. It is also, of course, perfectly conceivable that the target binding pocket is unrelated to the coding flank binding pocket.

Assuming that the hairpin inhibitor binds to the target binding pocket, our observation that it does not inhibit cleavage (hairpin formation) even when present at substantial molar excess is consistent with the third possibility, that the target binding site is revealed only after cleavage. Further studies of this inhibitor should allow us to answer more detailed questions about the nature of target binding.

### Implications for RAG-Mediated Transposition In Vivo

The initial discovery of RAG transposase activity prompted speculation that transposition could explain certain oncogenic translocations [2,5–8]. Thus far, however, there has been no direct evidence to implicate the transposition activity of the RAG proteins in any oncogenic chromosome rearrangements—nor has there been anything to guide our search for such evidence. We have now demonstrated that the RAG transposase exhibits striking target preferences, and that these preferences are largely modulated by target structure. Given that mammalian genomes contain a variety of structural elements, including palindromic sequences, triplet and tetra nucleotide repeat sequences capable of forming hairpin structures, and quadruplex structures that possess hairpin-type ends [29–33], there may be many preferred targets for the RAG transposase. In fact, many translocation breakpoints have been shown to lie at or near sites of DNA distortion [34–39]. The discovery of strong target preferences could serve as a useful guide to those searching for the footprint of RAG transposition in genomic rearrangements.

## Materials and Methods

### Proteins.

All experiments were performed using recombinant mouse RAG1 and RAG2; core RAG1 (residues 384–1,008) and either core (residues 1–387) or full-length RAG2 were expressed from the pEBG vector [40]. RAG1 and RAG2 were co-purified as glutathione-S-transferase fusion proteins from Chinese hamster ovary (RMP41) cells [41]. Recombinant human HMGB1 was purified from Escherichia coli as previously described [42].

### Oligonucleotide DNA substrates.

Precleaved 12-RSS oligonucleotide was generated by annealing DG10 to its complement [43]. Precleaved 23-RSS oligonucleotide was generated by annealing precleaved GSL61.1 (5′-CACAGTGGTAGTACTCTACTGTCTGGCTGTACAAAAACCCTGCAG) to its complement. Inverted repeat–encoding oligonucleotide sequences are as described in Figures 1A, S2A, and S2B. A double-stranded non-hairpin control identical in sequence to TA-2 was generated by annealing TA-2-top (5′-TAGCTCGAGACCTA) to TA-2-bottom (5′-TAGGTCTCGA). DR117TnRef was used as a reference oligonucleotide identical in sequence to the expected product of transposition of a 12-RSS into the AA hairpin tip (5′-CTGCAGGGTTTTTGTTCCAGTCTGTAGCACTGTGTTCCTGCAGCCCCAG). TA-2TnRef was used as a reference oligonucleotide identical to the expected sequence of the product of transposition of a 12-RSS into the TA-2 hairpin tip (5′-CTGCAGGGTTTTTGTTCCAGTCTGTAGCACTGTGTAGGTCTCGA). All radioactively labeled oligonucleotides were 5′-end labeled with P^32^.

### Plasmid DNA substrates.

Plasmid-encoded inverted repeats with a TA, GC, or CT tip (termed plasmids “TA,” “GC,” or “CT”) were generated by cloning a 106-bp inverted repeat into the EcoRI site of pUC8. For this, the two strands of the inverted repeat were synthesized to form a DNA fragment with a duplex EcoRI site and complementary single-strand region at the end that comprises the center of the inverted repeat. The strands were hybridized, cut with EcoRI, and ligated into EcoRI-cut pBR325. Following ligation, the plasmids were transformed into HB101 and clones selected for sensitivity to chloramphenicol. These were then transferred, as an EcoRI fragment, into pUC8. The resulting inverted repeats share a 51-bp repeat sequence (5′-pUC8 backbone-GAATTCCCAATTGATAGTGGTAAAACTACATTAGCAGACTGGGGCTGCAGG-dyad axis) and differ only in the identity of the four nucleotides at the dyad axis of the repeat, as specified in Figures 2–4. To confirm the central sequence of the inverted repeats, DNA samples were digested with AccI, BanI, or NheI for inverted repeats containing central TA, GC, or CT, respectively. Plasmids were purified by alkaline lysis and ethidium bromide CsCl gradient procedures. DNA topoisomers were made as described [44]. Plasmid A without a cruciform is pUC8; Plasmid A with a cruciform is F14C, a pUC8 plasmid containing a 106-bp inverted repeat cloned into the EcoRI site [45]. Plasmid B with GC-rich sequence is pJH452, a pJH290-related plasmid in which both the 12-RSS and 23-RSS have been removed by deletion of SalI and BamHI fragments, respectively [46,47]. Plasmid B without GC-rich sequence was obtained by removal of the ClaI fragment in pJH452. To place the 11-bp sequence 5′-GCCGCCGGGCG into the pUC8 backbone, an oligonucleotide containing the appropriate sequence flanked by EcoRI sites, 5′-TGACTGGAATTCGCCGCCGGGCGCCATGGGAATTCAATAGA, was annealed to its complement, digested with EcoRI, and cloned into the EcoRI site in pUC8.

### Oligonucleotide hairpin annealing.

Oligonucleotide-encoded inverted repeats were incubated at 100 °C for 9 min in 50 mM HEPES (pH 8.0) and 1 mM Ca^2+^ at a concentration of 0.5 pmol/μl and then transferred quickly to an ice water bath. Proper annealing of oligonucleotides into hairpins was determined by separating radiolabeled hairpins on a 16% native acrylamide gel and visualized by autoradiography (Figure S1).

### Oligonucleotide target transposition reactions.

Transposition reactions were carried out in vitro as described [15]. All reactions were performed using core RAG1 and either core RAG2 or full-length RAG2 as indicated. Briefly, 0.05 pmol of radiolabeled precleaved 12-RSS and 0.05 pmol of unlabeled precleaved 23-RSS were incubated with 100 ng each of RAG1 and RAG2, in addition to 25 ng of HMGB1, in buffer containing 5 mM Ca^2+^ at 37 °C for 20 min. A total of 0.5 pmol of target preparation was then added to reaction mix. In the case of inhibition reactions, 0.25 pmol (unless otherwise specified) oligonucleotide CT hairpin was mixed with 0.25-pmol target prior to target addition to reaction mix. Reactions were then incubated for 20 min at 24 °C. Mg^2+^ was added to a final concentration of 3 mM, and reactions were incubated for 30 min (unless otherwise specified) at 24 °C. Reactions were stopped by adding an equal volume of stop buffer containing 0.2% SDS and 0.35-mg/ml proteinase K in 100 mM Tris (pH 8.0) and 10 mM EDTA. For time course experiments, after Mg^2+^ addition, reaction was allowed to proceed until the appropriate time point before addition of an equal volume of stop buffer. Reaction products were separated on 12% or 15% denaturing acrylamide sequencing gels, and visualized by autoradiography.

### Plasmid target transposition reactions.

Transposition reactions were carried out in vitro as described above except that the entire reaction was carried out at 37 °C. A total of 200 ng of plasmid target was added to each reaction, except in Figure 3D, in which only 50 ng of target plasmid was added to each reaction. For inhibition reactions, 100 ng of plasmid target and 100 ng of plasmid-encoded CT cruciform were mixed prior to target addition. To digest transposition products, reaction products were sequentially extracted in phenol-chloroform and butanol, then ethanol precipitated in 300 mM sodium acetate prior to resuspension in appropriate digestion buffer. Following digestion, products were precipitated again and resuspended in alkaline loading buffer (50 mM NaOH, 1 mM EDTA, 3% Ficoll). Products were separated on native or denaturing 1% agarose gels, which were dried and visualized by autoradiography.

### Quantitation of reaction products.

After separation on a gel, radiolabeled products were detected using a Storm PhosphorImager (GE Healthcare, Little Chalfont, United Kingdom). Bands were quantified using ImageQuaNT software (GE Healthcare). Area under the curve measurements were used as band intensity values. Efficiency of transposition (percentage) was calculated as intensity of transposition products divided by the total intensity of labeled RSS. All error bars represent standard error of the mean.

### In vitro cleavage reactions.

The cleavage substrate was generated by PCR amplification of pJH290 using primers DR99 and DR100 [48,49]. Briefly, 0.13–0.20 pmol of PCR substrate was incubated in reaction buffer containing 5 mM Ca^2+^ with 100 ng each of RAG1 and RAG2, and 200 ng of HMGB1. This mixture was incubated for 15 min at 37 °C. MgCl_2_ was added to a final concentration of 5 mM, and the reaction was incubated for 3 h at 37 °C. The reaction was terminated by addition of an equal volume of stop buffer. Cleavage products were separated on a native 4%–20% Novex acrylamide gradient gel (Invitrogen, Carlsbad, California, United States) stained with vistra green, and visualized using a PhosphorImager (GE Healthcare). When CT oligonucleotide hairpin was added to the reaction, it was first mixed with cleavage substrate before addition of other reaction components (Figure 4D), or it was added after the 3-h reaction (Figure 5D).

### Physical analysis of target capture complexes.

Binding to hairpin targets was performed essentially as described [15]. Briefly, 0.13 pmol each of precleaved 12- and 23-RSS were incubated with 100 ng each of RAG1 and RAG2, and 80 ng of HMGB1 in 5.4 mM Ca^2+^ for 10 min at 37 °C. A mix of 0.5 pmol radiolabeled hairpin target and Mg^2+^ was added to the reaction such that the final concentration of Mg^2+^ was 4 mM. Reaction mixtures were incubated for 120 min at 37 °C and then placed on ice. Products were immediately resolved on a native 4%–20% Novex acrylamide gradient gel at 4 °C. Gels were then dried and visualized by autoradiography. When challenge with cold hairpin was performed, unlabeled hairpin (in an amount equimolar to radiolabeled target) was either mixed with radiolabeled target prior to addition to the reaction (Figure 5A and 5B), or added 120 min after addition of radiolabeled target/Mg^2+^ mix (Figure 5C).

The reaction conditions used to assess hairpin formation and transposition product disintegration are described in Protocol S1.

## Supporting Information

Figure S1 All Oligonucleotide Targets Fold and Remain in Hairpin Form (A) Oligonucleotide-encoded inverted repeats can engage in intra-strand annealing to form hairpins, or inter-strand annealing to form dimers.
(B) Oligonucleotide targets anneal intramolecularly. P^32^ 5′-end–labeled targets are folded into hairpins (see Materials and Methods) and then separated on a 16% native agarose gel at 4 °C.
(C) Hairpin oligonucleotides do not convert to dimeric species during the course of a transposition assay. At the end of a transposition assay using both labeled hairpin target and labeled signal ends, transposition products are separated on a 16% native agarose gel at 24 °C.
TG[hi], sample in which TG is prepared at a higher concentration (11.3 pmol/μl), such that substantial dimerization occurs.
(D) CT is refractory to RAG transposition, even though it is properly folded as a hairpin. Transposition products from C are separated on a 12% sequencing gel.
Oligonucleotides CT, TA, and TG are CT-2, TA-3, and TG-2 described in Figure S2A.
HP, hairpin; M1, TA-2BE, a blunt-ended oligonucleotide identical in sequence to TA-2; M2, TG-2 prior to annealing reaction, with both hairpin and dimer species; M3, TA-2TnRef, a reference oligonucleotide representing expected size of transposition products; No RAG, mock reaction lacking RAG proteins; SE, signal ends; TnP, transposition products.
(1.6 MB PDF)

Figure S2 Transposition Efficiency Is Unaffected by Hairpin Stem Sequence (A) Five oligonucleotide-encoded inverted repeats of different sequences are named according to the sequence of the two nucleotides just 5' of the dyad axis of the repeat; designation “-2” or “-3” for a particular hairpin indicates a different stem sequence from that tested in Figure 1.
(B) Diagram of a TA and a GC hairpin showing alterations in the nucleotide in the third position (n-3) from the dyad axis.
(C) Changes in stem sequence do not affect the target preferences of the RAG transposase. Transposition products formed from the five targets shown in (A) were separated on a 15% sequencing gel.
(D) Variations in the nucleotide at the third position from the dyad axis have only a small effect on targetability. Transposition products formed from targets shown in (B) were separated on a 15% sequencing gel. Laddering of transposition products is likely due to slight degradation of labeled signal end DNA.
M, TA-2TnRef, reference oligonucleotide representing the expected transposition product of TA-2; No RAG, mock reaction lacking RAG proteins; SE, signal ends; TnP, transposition products.
(4.0 MB PDF)

Figure S3 The Inhibitor Does Not Affect Hairpin Formation (A) Depiction of reaction: RAG proteins were mixed with a radiolabeled (star) oligonucleotide representing transposition product formed from a 12-RSS and a GC hairpin target, as well as a free, precleaved 23-RSS. Hairpin formation (transposition product disintegration) was assessed following addition of non-labeled inhibitor.
(B) Hairpin formation was not affected by addition of buffer only, CT hairpin, or GC hairpin. Hairpin products were separated on a 15% sequencing gel.
No RAG, mock reaction lacking RAG proteins.
(2.0 MB PDF)

Protocol S1 Supplemental Materials and Methods (35 KB DOC)

## Abbreviations

bp: base pair
RSS: recombination signal sequence
