# Supplementary material for: Target DNA Structure Plays a Critical Role in RAG Transposition
Source: PLoS Biol. 2006 Oct 24;4(11):e350. doi: 10.1371/journal.pbio.0040350 (PMC1618415; doi:10.1371/journal.pbio.0040350)
Supplement: Supplementary file 1 [file pbio.0040350.sg001.pdf]

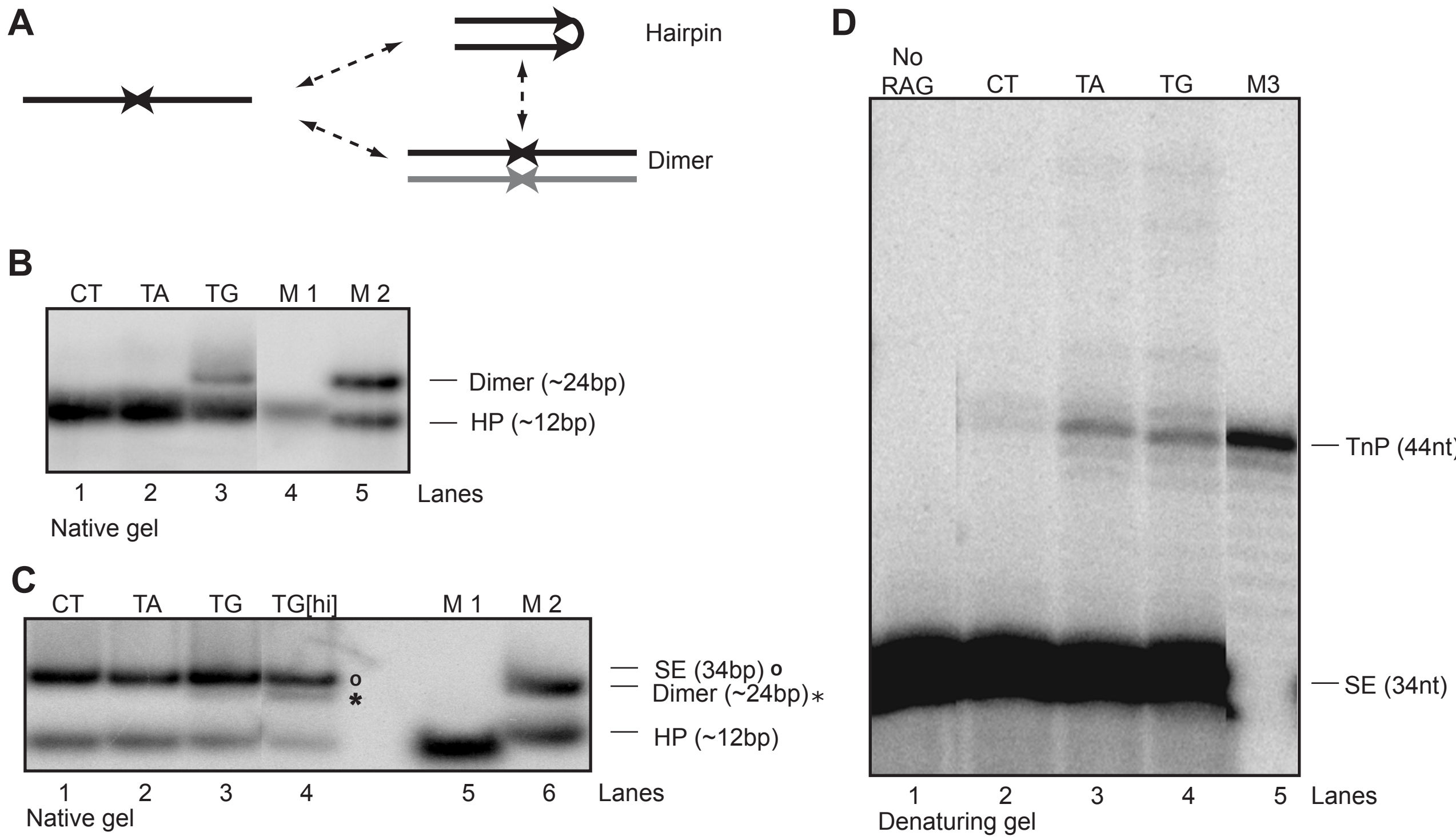
A
Hairpin
Dimer
B
CT TA TG M 1 M 2
Dimer (~24bp)
HP (~12bp)
1 2 3 4 5 Lanes
Native gel
C
CT TA TG TG[hi] M 1 M 2
SE (34bp) o
Dimer (~24bp) *
HP (~12bp)
1 2 3 4 5 6 Lanes
Native gel
D
No RAG CT TA TG M3
TnP (44nt)
SE (34nt)
1 2 3 4 5 Lanes
Denaturing gel
