# Supplementary material for: Target DNA Structure Plays a Critical Role in RAG Transposition
Source: PLoS Biol. 2006 Oct 24;4(11):e350. doi: 10.1371/journal.pbio.0040350 (PMC1618415; doi:10.1371/journal.pbio.0040350)
Supplement: Supplementary file 2 [file pbio.0040350.sg002.pdf]

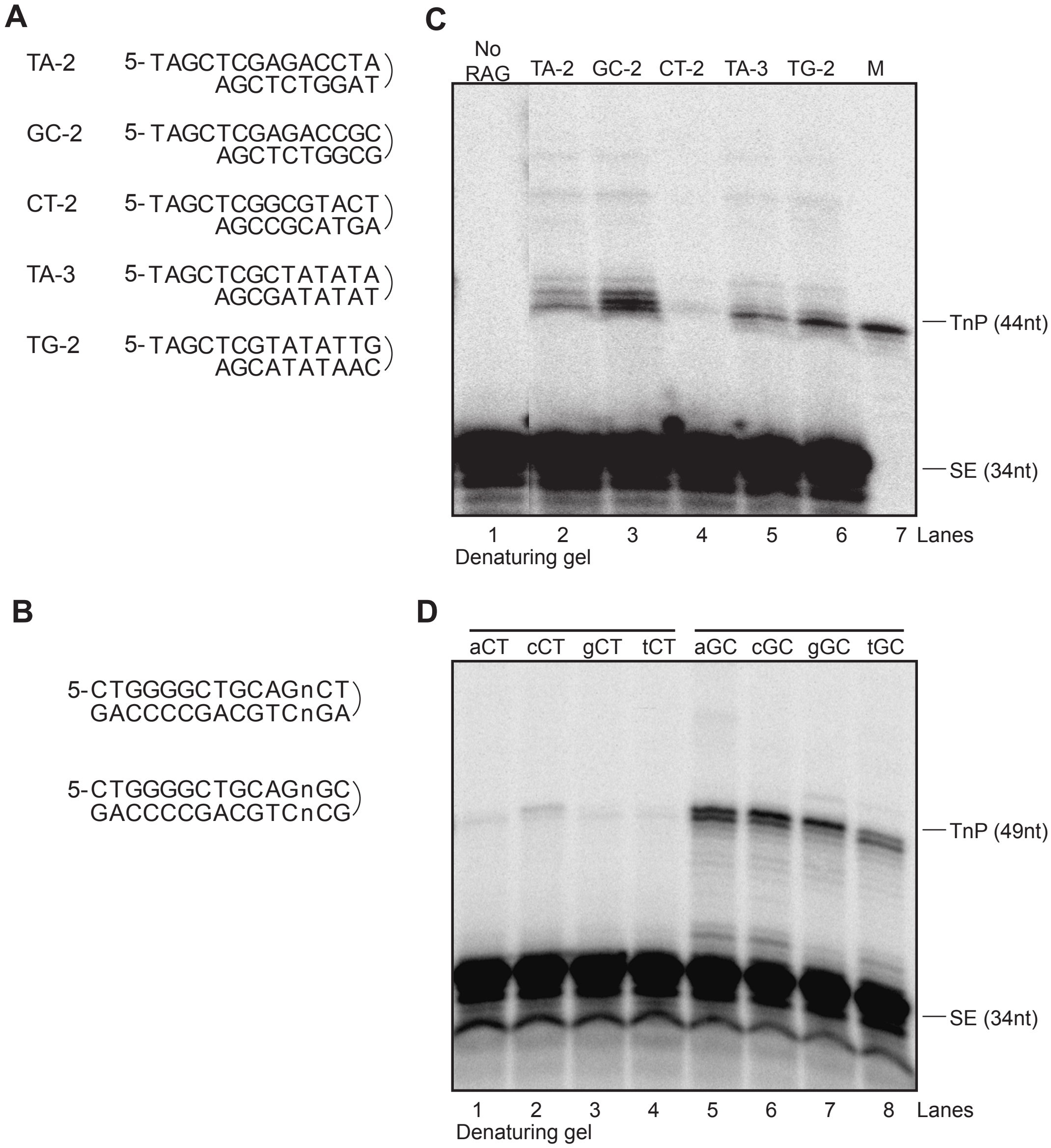
A
TA-2 5-TAGCTCGAGACCTA AGCTCTGGAT
GC-2 5-TAGCTCGAGACCGC AGCTCTGGCG
CT-2 5-TAGCTCGGCGTACT AGCCGCATGA
TA-3 5-TAGCTCGCTATATA AGCGATATAT
TG-2 5-TAGCTCGTATATTG AGCATATAAC
B
5-CTGGGGCTGCAGnCT GACCCCGACGTCnGA
5-CTGGGGCTGCAGnGC GACCCCGACGTCnCG
C
No RAG
TA-2
GC-2
CT-2
TA-3
TG-2
M
TnP (44nt)
SE (34nt)
1 2 3 4 5 6 7 Lanes
Denaturing gel
D
aCT cCT gCT tCT aGC cGC gGC tGC
TnP (49nt)
SE (34nt)
1 2 3 4 5 6 7 8 Lanes
Denaturing gel
