# Supplementary material for: Target DNA Structure Plays a Critical Role in RAG Transposition
Source: PLoS Biol. 2006 Oct 24;4(11):e350. doi: 10.1371/journal.pbio.0040350 (PMC1618415; doi:10.1371/journal.pbio.0040350)
Supplement: Supplementary file 3 [file pbio.0040350.sg003.pdf]

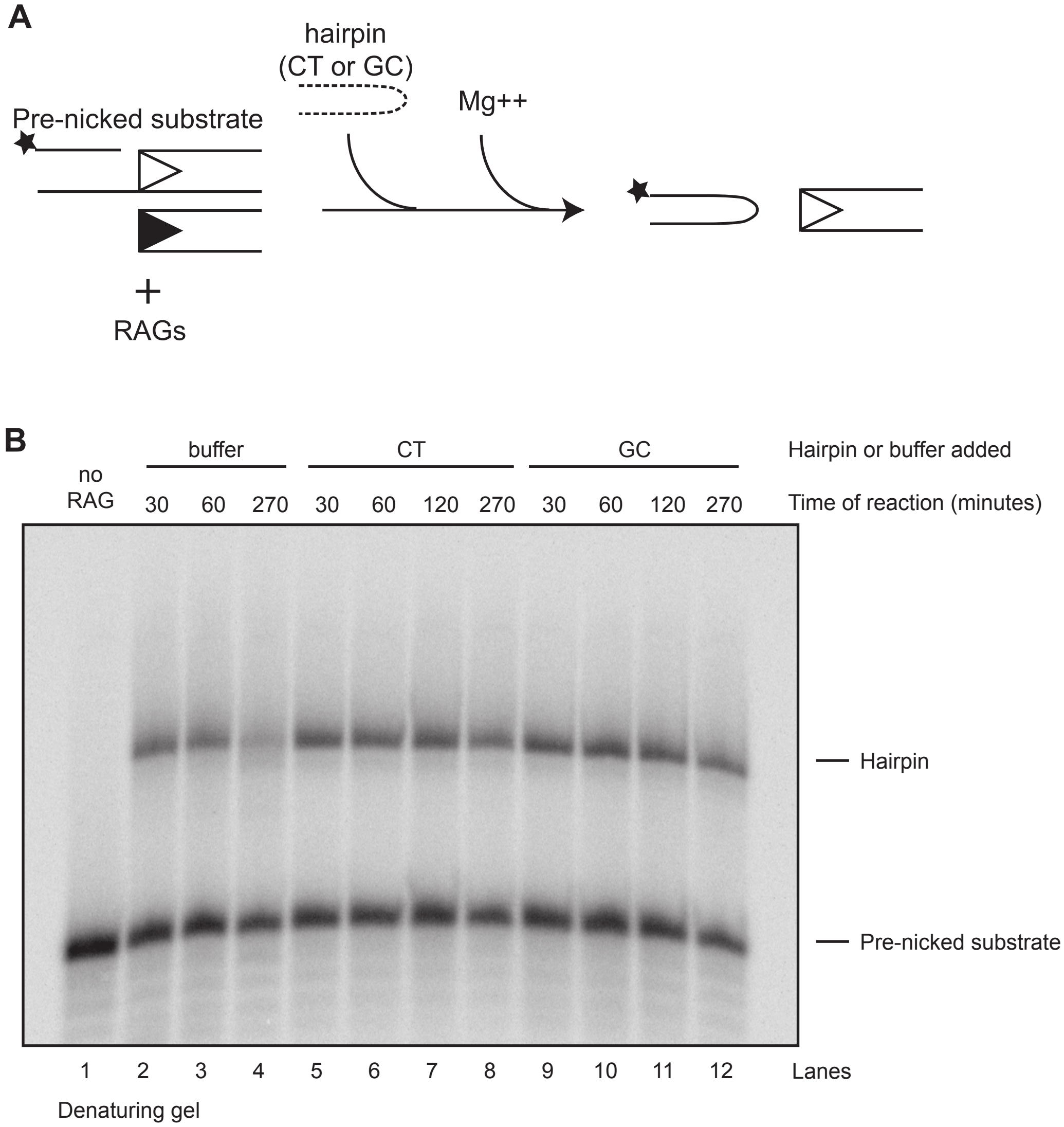
A
hairpin
(CT or GC)
Pre-nicked substrate
Mg++
+
RAGs
B
buffer
CT
GC
Hairpin or buffer added
no
RAG
30
60
270
30
60
120
270
30
60
120
270
Time of reaction (minutes)
Hairpin
Pre-nicked substrate
1
2
3
4
5
6
7
8
9
10
11
12
Lanes
Denaturing gel
