# Supplementary material for: Target DNA Structure Plays a Critical Role in RAG Transposition
Source: PLoS Biol. 2006 Oct 24;4(11):e350. doi: 10.1371/journal.pbio.0040350 (PMC1618415; doi:10.1371/journal.pbio.0040350)
Supplement: Supplementary file 4 [file pbio.0040350.sd001.doc]

**Protocol S1**

**Supplemental Materials & Methods**

**Hairpin formation assay**

For this reaction, pre-cleaved 23-RSS was formed by annealing GSL61.1 to its complement. The pre-nicked 12-RSS substrate was formed by annealing DG10 and TnAttack0a (5-CTGGGGCTGCAGGGC) to their complement TnAttack0b (5-CTGCAGGGTTTTTGTTCCAGTCTGTAGCACTGTGGCCCTGCAGCCCCAG). The hairpin formation assay was performed in reaction buffer identical to that of oligonucleotide target transposition reactions. Briefly, 0.05 pmol pre-cleaved 23-RSS and 0.05 pmol pre-nicked 12-RSS were incubated with 100 ng of each RAG and 25 ng HMGB1 in buffer containing 5 mM Ca^2+^ at 37°C for 20 minutes. 0.5 pmol of hairpin (either CT or GC) preparation was then added to reaction mix; reactions were then incubated for 20 minutes at 37°C. Mg^2+^ was added to a final concentration of 3 mM, and reactions were incubated for 30, 60, 120, or 270 minutes at 37°C. Reactions were stopped by adding an equal volume of formamide. Products were separated on a 15% sequencing gel.
